# Effects of Forchlorfenuron on the Morphology, Metabolite Accumulation, and Transcriptional Responses of *Siraitia grosvenorii* Fruit

**DOI:** 10.3390/molecules24224076

**Published:** 2019-11-11

**Authors:** Hongwu Shi, Jingjing Liao, Shengrong Cui, Zuliang Luo, Xiaojun Ma

**Affiliations:** Institute of Medicinal Plant Development, Chinese Academy of Medical Sciences and Peking Union Medical College, Beijing 100193, China; hwshi10@163.com (H.S.); ljj564293456@126.com (J.L.); c1061729635@163.com (S.C.)

**Keywords:** *Siraitia grosvenorii* fruit, CPPU, morphology, metabolites, gene expression

## Abstract

*Siraitia grosvenorii* fruit, called luo-han-guo (LHG), have been used as a traditional Chinese medicine (TCM) and dietary supplements for many years. Mogrosides, the main bioactive ingredients in LHG, are commercially available worldwide as a non-sugar-based and noncaloric sweetener. However, the production cannot meet the increasing market demand because of the low content of mogrosides and the small size of LHG. Therefore, some advanced technologies have been applied for improving the quality of LHG. Forchlorfenuron (CPPU), a plant growth regulator, is widely applied to promote plant yield and the secondary metabolite synthesis. Here, the content of nine mogrosides and three intermediates in LHG that were treated with three different concentrations of CPPU were determined by LC-MS/MS and GC-MS, respectively. The total content of mogrosides in LHG treated with CPPU was not enhanced, and the proportion of some main bioactive ingredients, including mogroside V (MV), were decreased relative to that of the control treatment. Morphological and cytological observations showed CPPU could make an early lignification in fruit epidermal cells, and 5 or 25 mg L^−1^ CPPU could inhibit LHG growth. The expression levels of 24 key genes in the mogroside biosynthesis pathway were measured and revealed that genes downregulated in upstream, and different expressions of *SgUGTs* would affect the accumulations and proportions of mogrosides in LHG induced by CPPU. This was the first study that applied CPPU individually on LHG, and assessed effects of CPPU on the morphology, the accumulation of metabolites, and expression profiles of 24 structural genes. The CPPU effects on LHG were undesirable, including development inhibition and the decrease of main mogroside content. These will provide guidance for the rational application of CPPU.

## 1. Introduction

*Siraitia grosvenorii* is a perennial vine of the Cucurbitaceae family, and its fruit, called Luo-Han-Guo (LHG) in Chinese, has been used as a traditional Chinese medicine (TCM) in treatment of colds, sore throats, and lung congestion, and also has been recognized as a safe flavor enhancer and sweetener in Japan, US, and New Zealand [1,2]. Mogrosides, the main bioactive components extracted from LHG, are zero-caloric and approximately 300 times sweeter than sucrose, which can be used as a sucrose substitute for diabetes and obesity patients [3]. Pharmacological studies have shown that mogrosides have many important effects, such as liver-protecting, antidiabetic, antioxidation, anti-inflammatory, antitumor, and hypoglycemic activities [4,5,6].

**Figure 1:**
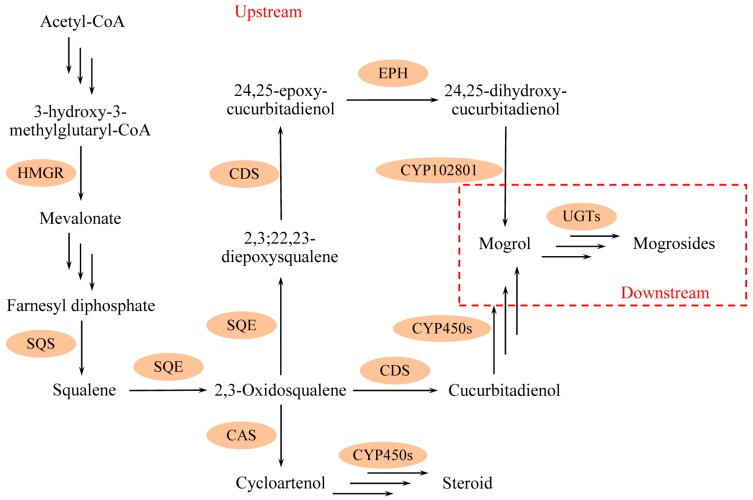
The diagram of the mogroside biosynthetic pathway. It was divided into upstream and downstream in this study. Acetyl-CoA is transformed into mogrol in the upstream by catalysis of HMGR (3-hydroxy-3-methyl glutaryl coenzyme A reductase), SQS (squalene synthase), SQE (squalene epoxidase), CDS (cucurbitadienol synthase), and CYP450 (cytochrome P450). Mogrol can be converted into different grades of mogrosides by UDP-glycosyltransferases. CAS (cycloartenol synthase) could translate 2,3-Oxidosqualene in to cycloartenol, which is the beginning of the steroid biosynthetic pathway. EPH, epoxide hydrolase; UGT, UDP-glycosyltransferases.

To date, key genes associated with mogroside biosynthesis have been completely cloned and characterized [7]. The mogroside biosynthesis pathway can be divided into upstream and downstream, as shown in Figure 1. In the upstream pathway, six enzyme families are considered for this biosynthetic process; acetyl-CoA is transformed to mogrol via catalysis by 3-hydroxy-3-methyl glutaryl coenzyme A reductase (HMGR), squalene synthase (SQS), squalene epoxidase (SQE), cucurbitadienol synthase (CDS), epoxide hydrolase (EPH), and cytochrome P450 (CYP450) [8]. Squalene is the precursor of 2,3-Oxidosqualene, and 2,3-Oxidosqualene can transform into cucurbitadienol under CDS catalysis. Cucurbitadienol, as a crucial triterpenoid skeleton, can be converted to a series of mogrosides. Cycloartenol, the starting point of synthesis of almost all plant steroids, competes for substrate 2,3-Oxidosqualene with the cucurbitadienol biosynthesis in LHG. In the downstream of the mogroside biosynthetic pathway, mogrol is transformed into different grades of mogrosides by UDP-glycosyltransferases (UGTs). Overall, twenty-four structural genes are considered in mogroside biosynthesis [9,10].

Mogrosides are the complex cucurbitane-type triterpenoid glycosides, and it is difficult to obtain these compounds by the chemical approach [11]. At present, commercially available mogrosides are mainly extracted from LHG. With an increased public awareness of healthy diets, the value of LHG extracts has been increasingly in the industry of TCM [12]. However, the low content of mogrosides in LHG cannot meet the increasing market demand. Market surveys showed that the larger the fruit, the higher the price because of the high content (1%–2%, *m*/*m*) of mogrosides in large LHG, and the extraction cost can be reduced by 1% when the mogroside content increases 0.1% [13]. Therefore, it is an urgent issue, taking some advanced technologies to achieve high-quality LHG.

Forchlorfenuron (CPPU, *N*-(2-Chloro-4-pyridyl)-*N*’-phenylurea), a synthetic cytokinin of the phenyl urea family, has been tested and applied to regulate fruit set, size, shape, and inflorescence morphology in *Torenia fournieri*, grapes, apples, pears, and melons [14,15,16,17,18,19]. It was approved for application to kiwi fruit and grape by the United States Environmental Protection Agency in 2009 [20]. CPPU is not only applied in agriculture, but also used with medicinal plants to improve the synthesis of secondary metabolites. For example, it can increase the content of total flavonoids and puerarin in kudzu roots [21], of catalpol and verbascoside in *Rehmannia glutinosa* tuber [22], and of schisandrol in *Schisandra chinensis* [23]. Moreover, CPPU has also been applied in combination with other plant growth regulators (PGRs) in fruit production. For example, CPPU applied with gibberellin can delay maturity, increase berry size, reduce anthocyanin content, and enhance firmness in grapes [24,25], and CPPU applied with paclobutrazol can improve the content of dry matter, protein, starch, and saponin in *Dioscorea opposita* root [26,27].

In this study, *S. grosvenorii* fruit at 20 days after flowering were treated with three different concentrations of CPPU, the determination of the transverse and vertical diameters of the fruit, and cytological observations of the fruit epidermis cell and pulp cell were performed, of which the results were applied to assess the morphological features of LHG after being treated with CPPU. In addition, the content of nine mogrosides and three intermediates in the fruit at different development stages were determined by high-performance liquid chromatography with tandem mass spectrometry (HPLC-MS/MS) or gas chromatography mass spectrometry (GC-MS). The expression levels of 24 structure genes involved in the mogroside biosynthesis were examined by a quantitative real-time PCR (qRT-PCR) experiment. The change in content and proportion of bioactive compounds were analyzed. The genes regulated by inducted CPPU will serve as a valuable reference for further studies. Overall, this is the first study that applied CPPU individually on LHG, and the effects on quality of LHG were investigated; these will provide guidance for the rational application of CPPU.

## 2. Results

### 2.1. Morphological and Cytological Observations

**Figure 2:**
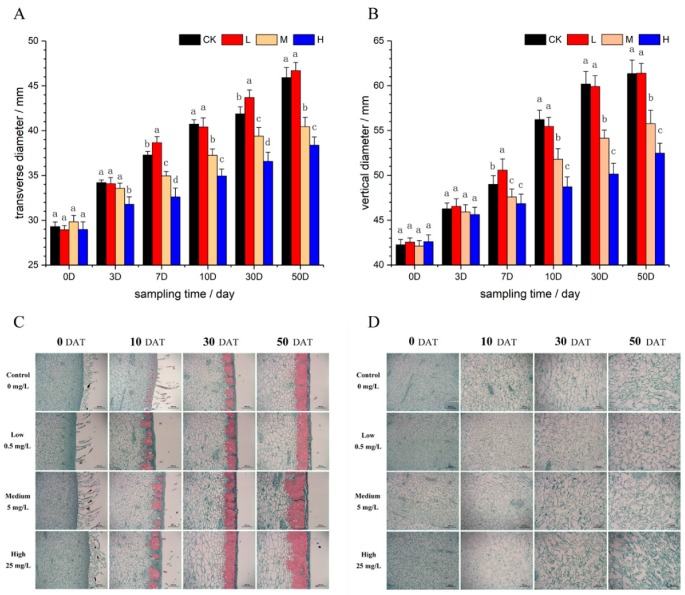
The size development and cytological observations of luo-han-guo (LHG). (**A**) and (**B**) display the changes of fruit size on transverse and vertical diameter, respectively. The CK, L, M, and H represent the control group, low-, medium-, and high-concentration forchlorfenuron (CPPU) treatment group, respectively. Different letters on the columns indicate significant difference at *p* < 0.05. (**C**) and (**D**) show cytological observations of the epidermis cells and pulp cells, respectively. Control, Low, Medium, and High mean the control group, low-, medium-, and high-concentration CPPU treatment group, respectively. DAT represents days-after-treatment.

The transverse and vertical diameters of LHG were measured from 0 to 50 days-after-treatment (DAT); the results are displayed in Figure 2 (2A and 2B). In the high-concentration CPPU treatment group, the fruit did not grow normally, and the transverse and vertical diameters were both smaller in these fruits than in the control fruit; about 0.83-fold and 0.85-fold smaller at 50 DAT, respectively. In the medium-concentration CPPU treatment, the fruit showed unnormal growth compared with the control fruit at 50 DAT, being 0.88-fold and 0.91-fold smaller in the transverse and vertical diameter, respectively. In addition, the fruit treated with the low concentration of CPPU did not significantly differ from the control fruit. Overall, it was obvious that the CPPU in medium- and high-concentrations inhibited fruit development. The inhibition started from 3 DAT for the transverse diameter development, as shown in Figure 2A, and 7 DAT for the vertical diameter development, as shown in Figure 2B. The results in the size of mature fruit and the starting day of inhibition indicated that the inhibition was more severe in the transverse diameter than the vertical diameter.

**Figure 3:**
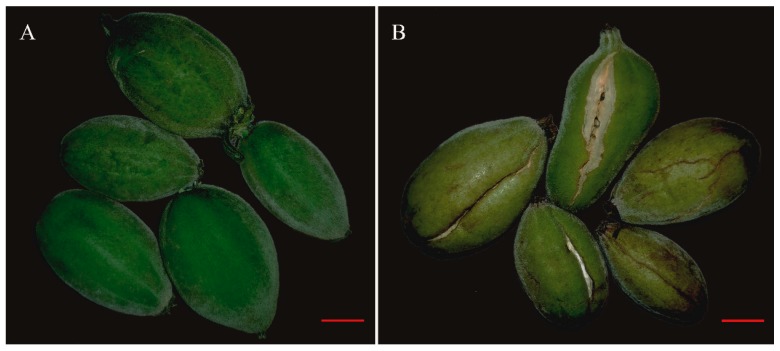
The morphological characteristics of *Siraitia grosvenorii* fruit at 7 DAT. (**A**) Control group, (**B**) the dehiscent *S. grosvenorii* fruit at 7 DAT with the medium-concentration CPPU treatment. Red scale bar, 1cm.

The epidermis cells and pulp cells in *S. grosvenorii* fruit were observed, which are exhibited in Figure 2C,D. Cytological observations showed that fruit fluff covered the fruit epidermis at 0 DAT (20 days after anthesis), and the dense cells in the fruit epidermis started to become lignified at 10 DAT (30 days after anthesis). The lignified cells connected to form a layer that became the hard fruit rind, as shown in Figure 2C. Interestingly, the epidermis cells of fruit treated with CPPU exhibited lignification earlier than that of the control group. In the high-concentration treatment, the fruit rinds were thicker than those of the control group at 50 DAT, and even the low-concentration group displayed some difference from the control group at 10 DAT. CPPU can promote *S. grosvenorii* fruit rind generation and make it hard. In addition, under medium CPPU concentrations, the cells under the epidermis were symmetrical and numerous at 10 DAT, and some dehiscent fruit were found at 7 DAT, as shown in Figure 3. We speculate that these cells divided rapidly under the epidermis while the epidermis partly lignified, which might have caused the dehiscent fruit. Microscopic observation of pulp cells revealed a dense distribution at 0 DAT and a continuous, rapid increase in cell number to 10 DAT; at this time, cell size in the CPPU treated groups was bigger than that in the control group, as shown in Figure 2D. Subsequently, the pulp cells continued to increase in size and became irregular shaped during 30–50 DAT. CPPU treatment had a little influence on pulp cell development; it is possible that the CPPU solution, after dipping the fruit, did not diffuse deeply into the fruit.

### 2.2. The Determination of Three Intermediates and Nine Mogrosides in S. grosvenorii Fruit

**Figure 4:**
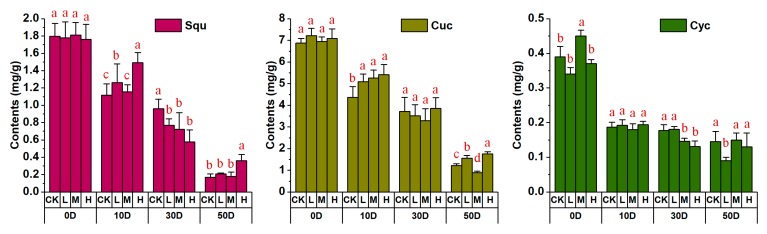
Effects of different concentrations of CPPU applied to LHG on the accumulation of aqualene, cucurbitadienol, and cycloartenol at four sampling times. Squ, Cuc, and Cyc represent squalene, cucurbitadienol, and cycloartenol. Different letters indicate significant differences at *p* < 0.05. CK, L, M, and H stand for control group, low-, medium-, and high-concentration CPPU treatment, respectively. D stands for the days after treatment.

In the upstream of the mogroside biosynthetic pathway, the content of the three compounds (squalene, cucurbitadienol, and cycloartenol) in *S. grosvenorii* fruit treated with different concentrations of CPPU were determined by GC-MS at different DAT, and the results exhibited obvious differences, as shown in Figure 4 and Appendix Table A1. The content of three intermediates decreased constantly in the control and treatment groups. On 50 DAT, the content of squalene showed a marked difference in the control and the high-concentration CPPU treatment groups (2.12-fold higher than that of the control). In the low- and medium-concentration CPPU groups, squalene content did not differ significantly from that in the control. Compared with the corresponding content in the control group on 50 DAT, the cucurbitadienol contents were 1.29-fold and 1.45-fold under low- and high-concentration treatments, respectively. The fruit in the medium-concentration treatment showed a 36% reduction in content of cucurbitadienol relative to the level in the control group on 50 DAT. Regarding content of cycloartenol, there was no significant difference between the medium- or high-concentration CPPU treatments and the control group, whereas it was decreased to 61% under the low concentration of CPPU. The rapid content decrease of the three intermediates might result from the efficient utilization of synthesis downstream. These results demonstrated that different concentrations of CPPU could affect the accumulation of compounds.

**Figure 5:**
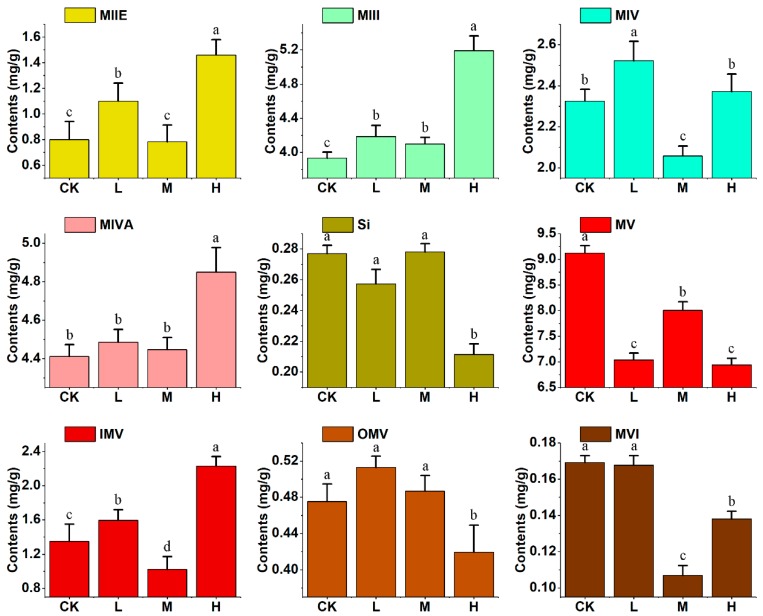
The content of nine mogrosides in 50 DAT. Different letters indicate significant differences at *p* < 0.05. CK, L, M, and H stand for control group, low-, medium-, and high-concentration CPPU treatment, respectively.

**Figure A1:**
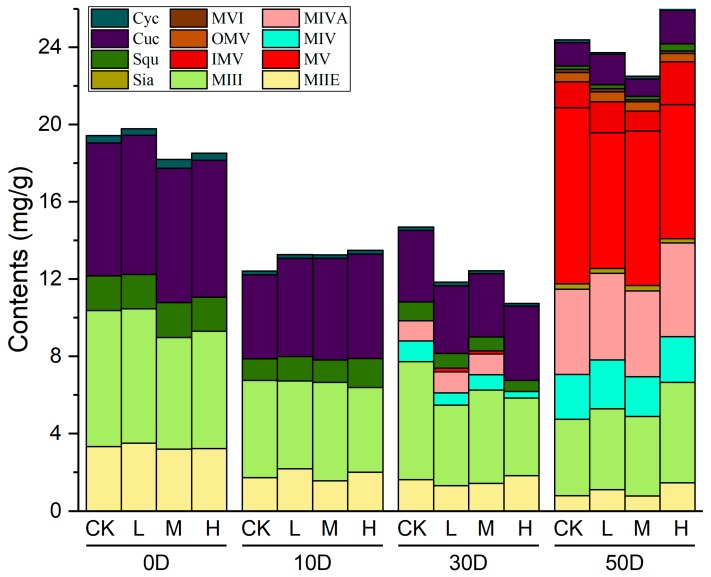
Graphical representation of the content of three intermediates and nine mogrosides, and the total content in the four sampling times. CK, L, M, and H stand for control group, low-, medium-, and high-concentration CPPU treatment, respectively.

Moreover, the contents of the nine mogrosides were determined by LC-MS/MS at different stages of fruit development, as shown in Appendix Figure A1 and Table A1. The mogroside V (MV) was detected (0.19 and 0.15 mg g^−1^, respectively) in the low- and medium-concentration CPPU groups in the 30 DAT, but not found in the control and high-concentration groups. The content of mogrosides MIIE and MIII were decreased during fruit development. Figure 5 displays the content of the nine mogrosides at 50 DAT. Compared with the content in the control group, the content of mogrosides MIIE, MIII, MIV, and IMV in the low-concentration CPPU treatment were increased (to 1.37-fold, 1.06-fold, 1.08-fold, and 1.18-fold, respectively), and the content of MV was decreased (to 0.77-fold). In the medium-CPPU treatment group, the content of mogroside MIII was slightly improved (to 1.04-fold) relative to the control content, whereas the content of mogrosides MIV, MV, IMV, and MVI were significantly reduced (to 0.88-fold, 0.87-fold, 0.76-fold, and 0.63-fold, respectively). Relative to the control treatment, the high concentration of CPPU greatly increased the content of mogrosides MIIE, MIII, MIVA, and IMV (to 1.82-fold, 1.32-fold, 1.10-fold, and 1.65-fold, respectively) but significantly decreased the content of Si, MV, OMV, and MVI (to 0.76-fold, 0.76-fold, 0.88-fold, and 0.82-fold, respectively). Overall, exogenous treatment with CPPU significantly promoted the accumulation of mogroside MIII and decreased the content of mogroside MV. The total content of mogrosides at 50 DAT in the control and low-, medium-, and high-concentration CPPU groups was 22.86, 21.86, 21.28, and 23.81 mg g^−1^, respectively. The total mogroside content in the high-concentration CPPU group had little difference compared with that of the control; the mogroside MV content proportion was 29.15% in this treatment group (vs. 39.89% in the control group), and 32.18% and 37.6% in the low- and medium-concentration CPPU group, respectively. The main active compound mogroside MV proportion was changed after CPPU treatment, which might affect the fruit’s flavor and its curative effects as a TCM.

### 2.3. Gene Expression Analysis

**Figure 6:**
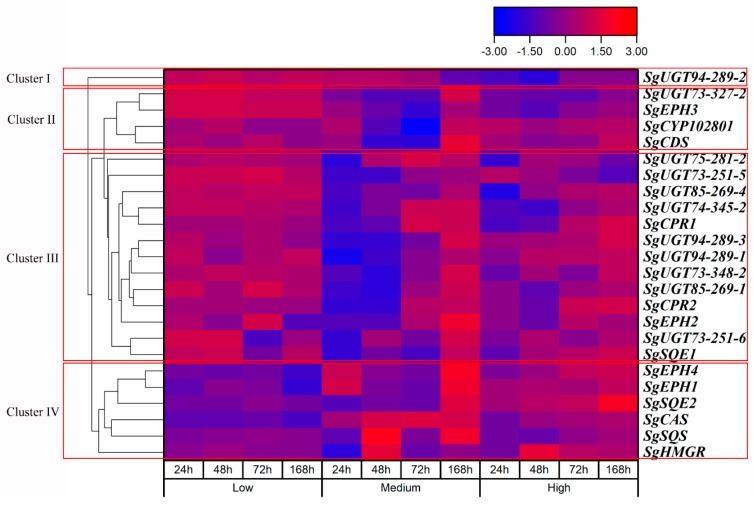
Hierarchical clustering analysis of the relative expression of 24 genes in LHG with three different CPPU concentration treatments. The genes with different expression levels were grouped into Cluster I, II, III, and IV.

Cluster analysis of the gene expression levels at the four sampling times and under the three treatment concentrations was performed, as shown in Figure 6. The expression levels of twenty-four key genes in the mogroside biosynthetic pathway treated with different concentrations of CPPU showed obvious differences. The genes with different expression levels were grouped into four main clusters. The gene *SgUGT94-289-2* was put into Cluster I, *SgUGT73-327-2*, *SgEPH*, *SgCYP102801*, and *SgCDS* were grouped into Cluster II, *SgUGT75-281-2*, *SgUGT73-251-5*, *SgUGT85-269-4*, *SgUGT74-345-2*, *SgCPR1*, *SgUGT94-289-3*, *SgUGT94-289-1*, *SgUGT73-348-2*, *SgUGT85-269-1*, *SgCPR2*, *SgEPH2*, *SgUGT73-251-6*, and *SgSQE1* were classified into Cluster III, the remaining genes including *SgHMGR*, *SgSQS*, *SgCAS*, *SgSQE2*, *SgEPH1*, and *SgEPH4* were categorized into Cluster IV. Relative to the control treatment, the genes in Cluster IV were upregulated in the fruit treated with low-concentration CPPU—these genes are significant in the upstream of the mogroside biosynthetic pathway—and genes in Cluster I, II, and III were upregulated, especially for the genes in the UGTs family. In the medium-concentration CPPU treatment group, most genes in Cluster III, especially the *SgUGTs* genes, were downregulated at 24 h or 48 h after treatment and then upregulated thereafter. As for the high-concentration CPPU treatment group, a handful of genes were downregulated at 24 h or 48 h after treatment, and a little upregulation was found in the latter sampling times; the gene *SgUGT94-289-2* was downregulated at all times. Overall, the expression levels of genes in Cluster III had obvious differences, upregulations were found in low-concentration CPPU, early downregulation and late upregulation in medium-concentration CPPU, and a little effect in high-concentration CPPU.

## 3. Discussion

*S. grosvenorii* is a perennial vine distributed in southern China in Guangxi. Some modern agricultural technologies, such as plant tissue culture [28,29], have been applied in the cultivation of *S. grosvenorii* plants; however, neither the content of mogrosides have markedly improved, nor a satisfactory fruit size was achieved. It is generally recognized that the production and distribution of the active compounds in medicinal plants is regulated by genetic factors, harvesting time, development environment, physiological factors, and chemical factors. It has been found that exogenous plant hormone, like methyl jasmonate, abscisic acid, and salicylic acid, could affect the accumulation of secondary metabolites in *Salvia miltiorrhiza* [30], *Glycyrrhiza uralensis* root [31], and *Uncaria tomentosa* [32]. Exogenous plant hormone applied in the *S. grosvenorii* might be an appropriate approach for good production, and Zhang et al. demonstrated that methyl jasmonate applied in different development stages of *S. grosvenorii* fruit significantly promotes the accumulation of squalene, cucurbitadienol, and mogrosides IIE [33].

A CPPU combined with gibberellin treatment achieved parthenocarpy in *S. grosvenorii* fruit in a previous study [34], whereas only a concentration CPPU (10 mg·L^−1^) treatment was performed and the achieved fruit size was unsatisfactory. In this study, the LHG treated with three different concentrations of CPPU displayed obvious differences in morphological characteristics and mogroside accumulations. The phenomenon that dehiscent fruit, found in the medium-concentration CPPU group at 7 DAT, and medium- and high-concentration of CPPU inhibited fruit development suggest that the inappropriate application of CPPU could lead to crop failure. Cytological observation revealed that LHG treated with CPPU presents lignification earlier, which would result in hard fruit rinds. The cells under the epidermis divided rapidly under CPPU treatment, and partial lignification of the epidermis might limit the space available for cell division. Research has indicated that CPPU has a cytokinin effect and that cell division is more sensitive to this effect than is cell enlargement [35,36]. Therefore, the combination of CPPU with other PGRs might be beneficial for *S. grosvenorii* fruit size development.

It is obvious that appropriate proportions of the ingredients are important for achieving a stable treatment effect in TCM. The active compounds in medicinal plants treated with exogenous plant hormone might be changed and their geo-herbal characteristics could not be guaranteed. In the *S. grosvenorii* fruit, the most important compounds are mogrosides, and mogroside MV content is regarded as an index of LHG quality and is required to be above 0.5% (*m*/*m*) in the Chinese Pharmacopoeia [37]. Our study found that the content and proportion of mogroside MV decreased after CPPU treatment, although those of some other mogrosides increased. Except for the usage as the materials in mogroside extraction production, LHG treated with CPPU should be carefully considered before use as a TCM. In addition, nine mogrosides have different sweetness, with sweetness decreasing in the order Si, IMV, MV, MIV, MIVA, MIII, MVI, OMV, and MIIE [38]; this means that the changes in the proportions of mogrosides under CPPU treatment might alter the fruit’s flavor and affect its dietary usage. Therefore, the effects of CPPU treatment on the quality of *S. grosvenorii* fruit, specifically, the proportions of mogrosides, should be investigated. Due to the limitation of little research of CPPU applied in medicinal plants, and lack of safety assessments, CPPU should be used cautiously in the cultivation application on *S. grosvenorii* used in herb crop production.

**Figure 7:**
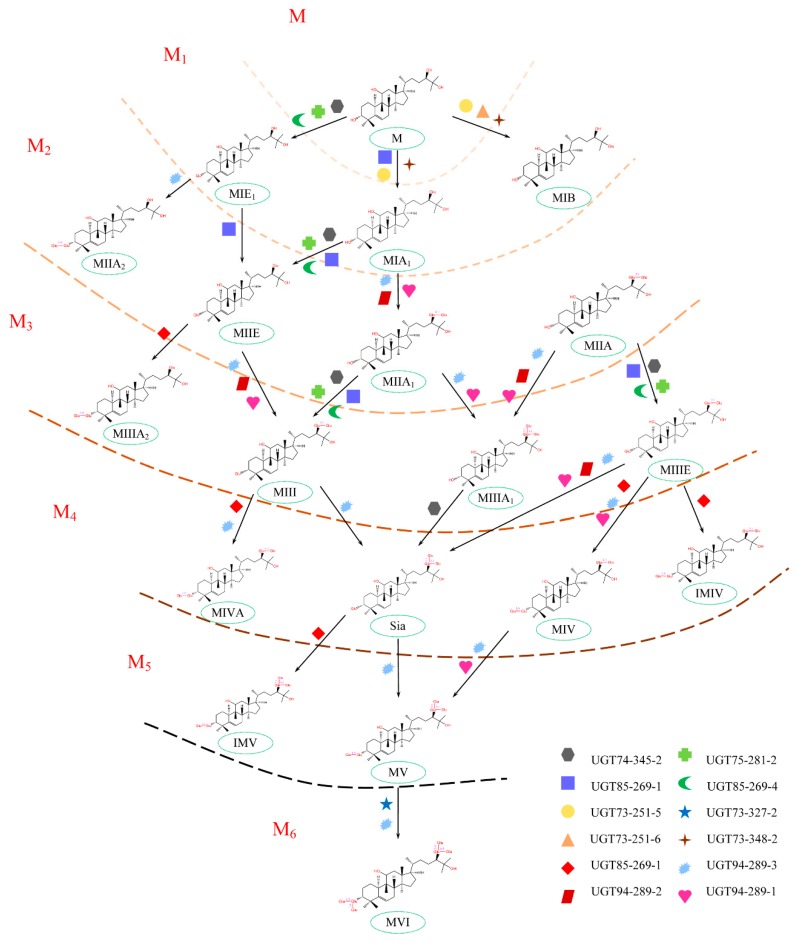
Twelve UGT synthases involved in the mogrol transformation into 18 mogrosides. The number in M1, M2, M3, M4, M5, and M6 stand for the number of β-d-glucopyranosyl conjugated with skeleton combined. “M” and “Sia” stand for “mogrol” and “siamenoside”, respectively. MIE1, MIA1, MIB, MIIA, MIIA1, MIIA2, MIIE, MIII, MIIIE, MIIIA1, MIIIA2, MIVA, MIV, IMIV, IMV, MV, and MVI represent mogroside IE1, mogroside IA1, mogroside IB, mogroside IIA, mogroside IIA1, mogroside IIA2, mogroside IIE, mogroside III, mogroside IIIE, mogroside IIIA1, mogroside IIIA2, mogroside IVA, mogroside IV, isomogroside IV, isomogroside V, mogroside V, and mogroside VI, respectively.

The biosynthetic pathway of mogrosides has been studied extensively, and most of the related genes have been identified. In the upstream of the biosynthetic pathway, as shown in Figure 1, the number of genes encoding SQE, EPH, and CPR (cytochrome P450 reductase) are 2, 4, and 2, respectively. In this study, *SgSQE2*, *SgEPH1*, and *SgEPH2* were put into Cluster IV; downregulation or little impact found in LHG treated with different concentrations of CPPU suggest that these genes have different functions and might be regulated in other mechanisms. In another sides, *SgSQE1*, *SgEPH2*, *SgCPR1*, and *SgCPR2* were gathered in Cluster III and upregulated. *SgCPR* is the redox partner of various *SgCYP450s* involved in secondary metabolism [39], its upregulation can play a significant role in the promotion of the mogrol accumulation, which would further improve mogroside accumulation downstream in this study. Genes in Cluster IV were almost downregulated in the low-concentration CPPU treatment, which would hinder the generation and conversion of intermediates upstream. In the downstream of the biosynthetic pathway, as shown in Figure 7, it has been identified that twelve genes in the UGT family participate in the process of mogrol converting into mogrosides [10]. Gene expression in this study displayed that nine genes in Cluster III (including *SgUGT73-251-5*, *SgUGT73-251-6, SgUGT73-348-2, SgUGT74-345-2, SgUGT75-281-2*, *SgUGT85-269-1, SgUGT85-269-4*, *SgUGT94-289-3*, and *SgUGT94-289-1*) were upregulated, and the expression of genes in the *SgUGT73* (*SgUGT73-251-5*, *SgUGT73-251-6, SgUGT73-348-2*) family were responsible for the mogroside M1 (MIA1 and MIB) biosynthesis. *SgUGT74-345-2, SgUGT75-281-2*, and *SgUGT85-269-4* could affect different grades of mogroside biosynthesis because of their UGT catalytic function on the C3 of the mogroside skeleton [7]. The UGT85-269-1 could make another β-d-glucopyranosyl conjugate with the β-d-glucopyranosyl on C3 of the skeleton. UGT94-289-3 and UGT94-289-1 were unspecific for the mogroside biosynthesis, especially UGT94-289-3, which can transform mogroside IIE into mogroside V. Overall, it is difficult to analyze the correlation between mogroside accumulation and the level of relative gene expression in plant development because of the intricate network of biosynthetic pathways.

In this study, the proportions of mogrosides were markedly affected by CPPU treatment, especially that of MV, which might have been due to the changes of gene expression in the biosynthetic pathway. In the low-concentration CPPU treatment group, *SgSQE1* and *SgCDS* were upregulated and cucurbitadienol content was increased, whereas *SgHMGR* and *SgSQS* were inhibited. CPPU treatment might inhibit squalene biosynthesis; although the downstream genes were upregulated, a lack of pre-substrates would affect mogroside biosynthesis. Under high-concentration CPPU treatment, squalene and cucurbitadienol accumulated significantly, and some genes were slightly upregulated at 48 h after treatment, and the proportions of mogrosides were changed. The structural genes in the mogroside downstream pathway are not specifically for biosynthesis of individual compounds, especially the *SgUGTs*. It demonstrated that genes are up/downregulated by CPPU treatment, which will serve as a valuable reference for further studies on the regulation of gene expression in the mogroside biosynthetic pathway.

## 4. Materials and Methods

### 4.1. Chemicals and Reagents

Nine mogroside reference compounds, comprising mogroside IIE (MIIE, purity 98.47%), mogroside III (MIII, purity 99.30%), mogroside IV (MIV, purity 98.57%), siamenoside I (Si, purity 99.45%), mogroside IVA (MIVA, purity 98.77%), mogroside V (MV, purity 99.26%), iso-mogroside V (IMV, purity 98.08%), 11-*O*-mogroside V (O-MV, purity 99.69%), and mogroside VI (MVI, purity 91.07%), were purchased from Chengdu Must Bio-Technology Co., Ltd. (Sichuan, China). The standard substances of squalene and cycloartenol were purchased from the National Institutes for Food and Drug Control (Beijing, China), and cucurbitadienol was provided by the Chinese Academy of Agricultural Science (Beijing, China). HPLC-grade methanol, formic acid, acetonitrile, and *n*-hexane were obtained from Fisher (Emerson, IA, USA). Other analytical-grade reagents were purchased from Sinopharm Chemical Regent Beijing (Beijing, China). The Milli-Q purification system (Millipore, MA, USA) provided the deionized water.

### 4.2. Plant Materials, CPPU Treatments, and Sample Preparation

Twenty *S. grosvenorii* plants grown under identical natural conditions were selected in Guilin City, Guangxi, China (24°57′36.16” N, 110°02′19.67” E). The control group and the treatment groups each comprised five plants. For the treatment groups, *S. grosvenorii* fruit at 20 days after flowering were dipped in CPPU solution at one of three concentrations (low concentration: 0.5 mg L^−1^, medium concentration: 5 mg L^−1^, and high concentration: 25 mg L^−1^) for approximately 30 s until the surface was uniformly wetted. Ethanol was used as a cosolvent for the preparation of the CPPU stock solution with a final concentration of 2% (*v*/*v*), and 2% (*v*/*v*) ethanol was used also for the control group. Samples were collected at different time points (0, 1, 2, 3, 7, 10, 30, and 50 DAT), and five biological replicates were obtained for each sample. Additionally, the fruit transverse diameter and vertical diameter were measured by an Absolute Digital Caliper (Mitutoyo, Tokyo, Japan) before treatment and sampling. The samples collected at 0, 1, 2, 3, and 7 DAT were cut into small pieces, immediately frozen in liquid nitrogen, and stored at -80 °C until further processing.

The dry powdered sample (0.5 g) was accurately weighted and suspended in 25 mL of methanol/water (80:20, *v*/*v*) with KOH (5%, *m*/*v*), then the sample was sonicated in an ultrasonic water bath operating at 40 kHZ with an output power of 300 W for 1 h at room temperature. Duplicate extraction was required and both extracts were mixed and then diluted to 100mL with methanol/water (80:20, *v*/*v*), and finally, the sample solution was filtered through a 0.22 μm polyvinylidene difluoride filter into 1.5 mL glass vials for LC-MS/MS determination. For the analysis of the nonpolar compounds—squalene, cucurbitadienol, and cycloartenol—from LHG, 50 mL of the above mentioned methanol/water extracts was extracted three times with the same volume of *n*-hexane. The combined extracts were dried under reduced pressure distillation and dissolved in 1 mL of *n*-hexane and then GC-MS analyzed.

### 4.3. Morphological and Cytological Observations

The fruit samples collected at 0, 10, 30, and 50 DAT were fixed in FAA (formalin, acetic acid, and alcohol) solution from Coolaber Biotech (Beijing, China), containing 45% ethanol, formaldehyde, and acetic acid (90:5:5 by volume). Then, the samples were softened and dehydrated. Subsequently, a thick section was cut from each sample and embedded in paraffin, and a transverse 10 μm thick slice was removed from the section by microtome (Leica Microsystems SM2500, Wetzlar, Germany). The slice was mounted and stained with safranin-fast green. Finally, photos were taken under a high-resolution digital microscope (Leica DM2500; Leica Microsystems GmbH, Wetzlar, Germany).

### 4.4. GC-MS Analysis of Squalene, Cucurbitadienol, and Cycloartenol

**Figure A2:**
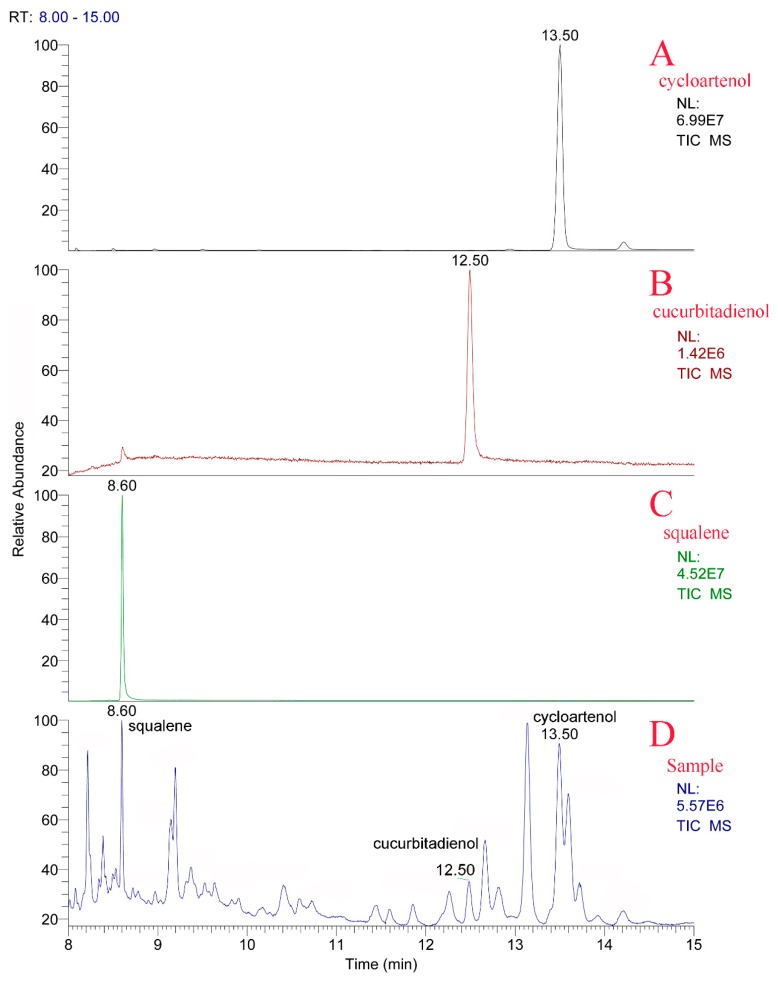
Typical GC-MS total ion chromatograms. (**A**), (**B**), and (**C**) is cycloartenol, cucurbitadienol, and squalene standards, respectively. (**D**) Examined sample.

GC-MS was performed on a Thermo Scientific ISQ Single Quadrupole GC-MS (Thermo Scientific, Waltham, MA, USA). Three intermediates (squalene, cucurbitadienol, and cycloartenol) were measured. The fruit samples for GC-MS analysis were prepared and the basic operational conditions were as described previously [13,33]. Helium gas was used as the carrier gas, and the carrier flow rate was 1.5 mL min^−1^. The initial oven temperature was 160 °C and was held for 1 min, then gradually increased to 300 °C at a rate of 20 °C·min^−1^ and then held for 10 min at 300 °C. A sample of 10 μL was injected automatically into the monitor system in 1:10 split mode. Selective ion monitoring mode (ions selected for scanning: *m*/*z*: 426, 410, 408, 393, 341, 367, 274, 95, 69) was chosen. Additionally, identification of the three components was performed by matching the fragment ion peak and retention time with standards, as shown in Appendix Figure A2.

### 4.5. LC-MS/MS Analysis of Nine Mogrosides

**Figure A3:**
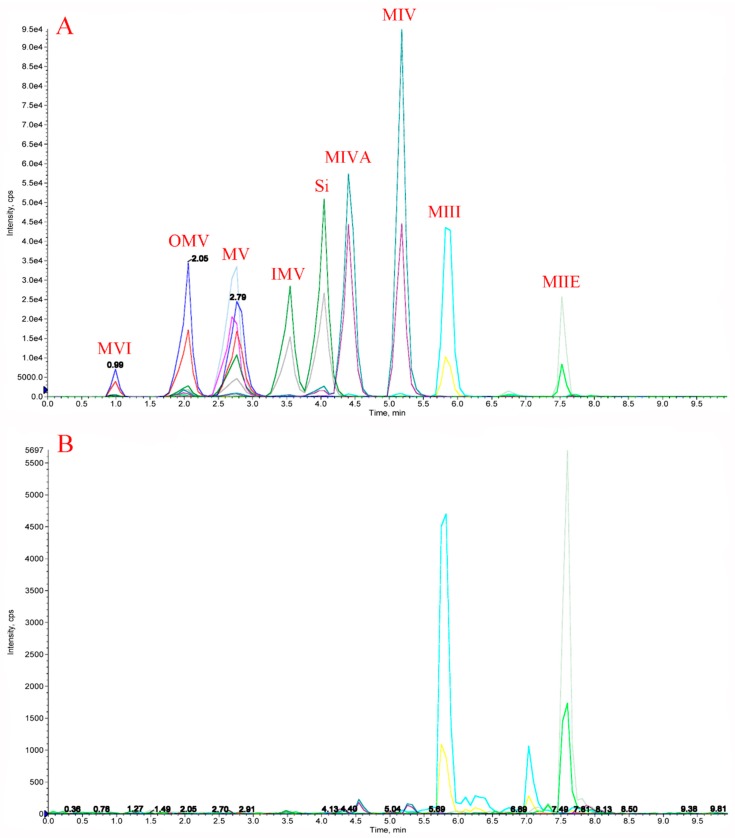
Typical LC-MS/MS total ion chromatograms. (**A**) Standard solutions of nine analytes. (**B**) Real sample. MVI, OMV, MV, IMV, MIVA, MIII, MIIE stand for mogroside VI, 11-oxo-mogroside V, mogroside V, isomigroside V, mogroside IVA, mogroside III, and mogroside IIE, respectively.

Mogroside MIIE, MIII, MIV, MIVA, Si, MV, IMV, OMV, and MVI were detected by LC-MS/MS following previously described methods [38]. The HPLC system consisted of an Agilent Technologies 1260 Series LC system (Agilent, USA) equipped with an automatic degasser, a quaternary pump, and an autosampler. Chromatographic separations were performed on an Agilent Poroshell 120 SB C18 column (100 × 2.1 mm, 2.7 μm) by gradient elution using a mobile phase consisting of (A) water (containing 0.1% formic acid) and (B) acetonitrile (containing 0.1% formic acid) with the following gradient procedure: 0 min, 26% B; 5 min, 32% B; 5.01–5.50 min, 80% B; and 5.51–10.0 min, 26% B. The flow rate was 0.25 mL·min^−1^, and the injection volume was 10 μL. The column effluent was monitored using a 4500 QTRAP^®^ LC-MS/MS system (AB Sciex, Toronto, Canada). The compound-dependent instrumental parameters were optimized and listed in Appendix Table A2. Both the standards and samples of nine mogrosides in the LC-MS/MS chromatograms are displayed in Appendix Figure A3.

### 4.6. Quantitative Real-Time PCR (qRT-PCR) Analysis of Gene Expression

Twenty-four structural genes in the mogroside biosynthetic pathway were measured by qRT-PCR. Total RNA extraction, quality and concentration assessment, and the expression profiles of the 24 structural genes determined using the BIO-RAD CFX system (BIO-RAD, USA) were performed as described previously [33]. First-strand cDNA was synthesized by TransScript One-Step gDNA Removal and cDNA Synthesis SuperMix (Transgen Biotech, Beijing, China). The primer sequences used in this experiment are listed in Appendix Table A3. *S. grosvenorii* ubiquitin (*SgUBQ*) was used as an internal reference gene [40]. Relative expression levels of each sample were calculated using the 2-ΔΔCt method [41].

### 4.7. Statistical Analysis

All results were presented as mean ± standard deviation (SD) of the number of experiments. The statistical significances of the fruit size change, concentration values, as well as gene expression levels were tested using Student’s *t* test implemented in the SPSS software (version 20.0, IBM, Chicago, IL, USA). The heatmap of hierarchical clustering was generated with Origin 2017 (OriginLab Co., Northampton, MA, USA).

## 5. Conclusions

In this study, the content of nine active components and three intermediates in LHG treated with different concentrations of CPPU were determined by LC-MS/MS and GC-MS, respectively. The results displayed that the total content of mogrosides in LHG treated with CPPU was not significantly different, and the proportional decrease of the main active compound mogroside MV was found in all treatment groups. Morphological and cytological observations showed the medium- or high-concentration CPPU could inhibit LHG development, and CPPU could affect the LHG rind generation. Twenty-four key gene expression levels revealed that genes downregulated in upstream and different expressions of *SgUGTs* would affect the accumulations and proportions of mogrosides. This study provides basic data of CPPU effects on the morphological characteristics, mogroside accumulation, and relative gene expression. Some undesirable consequences from LHG treated with CPPU should be concerning, and the application of CPPU should be cautious in the cultivation of *S. grosvenorii* used in herb crop production.

## Abbreviations

LHG	Luo-Han-Guo
TCM	Traditional Chinese Medicine
CPPU	Forchlorfenuron
DAT	Days-after-treatment
HMGR	3-hydroxy-3-methyl glutaryl coenzyme A reductase
SQS	squalene synthase
SQE	squalene epoxidase
CDS	cucurbitadienol synthase
EPH	epoxide hydrolase
CYP450	cytochrome P450
UGT	UDP-glycosyltransferases
PGR	plant growth regulators

## Appendix A

**Table A1:** Contents (mg g^−1^) of 12 compounds in tested samples (*n* = 5, mean ± SD).

**Compounds**	**CK (Control)**			
	**0D**	**10D**	**30D**	**50D**
Squalene	1.80 ± 0.15	1.12 ± 0.13	0.96 ± 0.11	0.17 ± 0.04
Cucurbitadienol	6.87 ± 0.22	4.36 ± 0.49	3.71 ± 0.65	1.21 ± 0.09
Cycloartenol	0.39 ± 0.03	0.19 ± 0.01	0.18 ± 0.02	0.14 ± 0.03
MIIE	3.33 ± 0.07	1.73 ± 0.06	1.63 ± 0.16	0.80 ± 0.14
MIII	7.05 ± 0.20	5.02 ± 0.09	6.09 ± 0.21	3.93 ± 0.07
MIV	ND ^a^	ND	1.09 ± 0.01	2.33 ± 0.06
MIVA	ND	ND	1.04 ± 0.04	4.41 ± 0.06
Si	ND	ND	ND	0.28 ± 0.01
MV	ND	ND	ND	9.12 ± 0.15
IMV	ND	ND	ND	1.35 ± 0.20
OMV	ND	ND	ND	0.48 ± 0.02
MVI	ND	ND	ND	0.17 ± 0.00
**Compounds**	**Low Concentration Treatment**			
	**0D**	**10D**	**30D**	**50D**
Squalene	1.78 ± 0.18	1.26 ± 0.22	0.77 ± 0.08	0.21 ± 0.01
Cucurbitadienol	7.21 ± 0.35	5.09 ± 0.35	3.51 ± 0.51	1.56 ± 0.12
Cycloartenol	0.34 ± 0.02	0.19 ± 0.02	0.18 ± 0.01	0.09 ± 0.01
MIIE	3.40 ± 0.11	2.18 ± 0.01	1.31 ± 0.02	1.10 ± 0.14
MIII	6.96 ± 0.25	4.54 ± 0.11	4.16 ± 0.27	4.18 ± 0.13
MIV	ND	ND	0.63 ± 0.03	2.52 ± 0.09
MIVA	ND	ND	1.09 ± 0.08	4.49 ± 0.07
Si	ND	ND	ND	0.26 ±0.01
MV	ND	ND	0.19 ± 0.03	7.03 ± 0.14
IMV	ND	ND	ND	1.60 ± 0.12
OMV	ND	ND	ND	0.51 ± 0.01
MVI	ND	ND	ND	0.17 ± 0.01
**Compounds**	**Medium Concentration Treatment**			
	**0D**	**10D**	**30D**	**50D**
Squalene	1.81 ± 0.14	1.16 ± 0.08	0.72 ± 0.19	0.18 ± 0.05
Cucurbitadienol	6.95 ± 0.21	5.26 ± 0.37	3.29 ± 0.55	0.89 ± 0.07
Cycloartenol	0.45 ± 0.02	0.18 ± 0.02	0.15 ± 0.01	0.15 ± 0.02
MIIE	3.20 ± 0.19	1.56 ± 0.12	1.42 ± 0.10	0.78 ± 0.13
MIII	5.78 ± 0.54	5.10 ± 0.01	4.83 ± 0.25	4.10 ± 0.08
MIV	ND	ND	0.79 ± 0.05	2.06 ± 0.05
MIVA	ND	ND	1.07 ± 0.05	4.45 ± 0.06
Si	ND	ND	ND	0.28 ± 0.01
MV	ND	ND	0.15 ± 0.01	8.00 ± 0.16
IMV	ND	ND	ND	1.02 ± 0.15
OMV	ND	ND	ND	0.49 ± 0.02
MVI	ND	ND	ND	0.11 ± 0.01
**Compounds**	**High Concentration Treatment**			
	**0D**	**10D**	**30D**	**50D**
Squalene	1.76 ± 0.17	1.49 ± 0.11	0.58 ± 0.14	0.36 ± 0.07
Cucurbitadienol	7.09 ± 0.44	5.42 ± 0.47	3.85 ± 0.50	1.75 ± 0.10
Cycloartenol	0.37 ± 0.01	0.19 ± 0.01	0.13 ± 0.02	0.13 ± 0.04
MIIE	3.22 ± 0.07	2.00 ± 0.04	1.83 ± 0.05	1.46 ± 0.12
MIII	6.08 ± 0.31	4.38 ± 0.05	4.00 ± 0.05	5.19 ± 0.17
MIV	ND	ND	0.34 ± 0.03	2.37 ± 0.08
MIVA	ND	ND	ND	4.85 ± 0.13
Si	ND	ND	ND	0.21 ± 0.01
MV	ND	ND	ND	6.94 ± 0.13
IMV	ND	ND	ND	2.23 ± 0.11
OMV	ND	ND	ND	0.42 ± 0.03
MVI	ND	ND	ND	0.14 ± 0.00

^a^ Not detected.

**Table A2:** MS parameters of nine mogrosides in LC-MS/MS determination experiment.

Analytes	Molecular Formula	t_R_ (min)	*m*/*z* Precursor	*m*/*z* Productions	DP (V)	CE (eV)
MIV	C_66_H_112_O_34_	1.16	1447.6	1285.4 ^a^/1123.5	−230	−105
OMV	C_60_H_100_O_29_	2.62	1283.7	1121.6 ^a^/959.5	−220	−90
MV	C_60_H_102_O_29_	3.78	1285.4	1123.5 ^a^/961.4	−220	−85
IMV	C_60_H_102_O_29_	4.64	1285.4	1123.5 ^a^/961.4	−210	−85
Si	C_54_H_92_O_24_	5.35	1123.4	961.6 ^a^/799.2	−220	−75
MIVA	C_54_H_92_O_24_	5.91	1123.4	961.6 ^a^/799.2	−220	−75
MIV	C_54_H_92_O_24_	7.01	1123.4	961.6 ^a^/799.2	−220	−75
MIII	C_48_H_82_O_19_	7.82	961.4	799.5 ^a^/637.2	−170	−70
MIIE	C_42_H_72_O_14_	8.11	799.5	637.6 ^a^/475.5	−170	−65
Source temperature (°C)		500				
Ionization voltage (V)		−4500				
GS1 (psi)		55				
GS2 (psi)		55				
CUR (psi)		20				
CAD		high				
Dwell time (ms)		400				
EP (V)		−10				
CXP (V)		−10				

^a^ product ion used for quantification. Abbreviations: t_R_: retention time. DP: declustering potential; CE: collision energy; CAD: collision activation dissociation; EP: entrance potential; CXP: collision cell exit potential.

**Table A3:** Primers used in the qRT-PCR experiment.

Gene Name	The Forward Primer Sequence (5′-3′)	The Reverse primer Sequence (5′-3′)
*SgHMGR*	TAGGCTCCAAAGTATCCG	CAGTTTACAGCAGCAGGTT
*SgSQS*	CTGAGACACCCA GATGACT	GAGGGCTCGCAGAACAAGA
*SgSQE1*	GCTTCGACCATCAACACATTG	TTCCTCCAAGGCTCAAGTAATC
*SgSQE2*	GCCCACTCTCAGCAAGTATC	GAAGATTCCTCCGAGGCTTAAA
*SgCDS*	GTTGGGTTGAAGATCCCTACTC	CCACAACTGGCTCCCATTAT
*SgCAS*	CAAATACAACATGCTCACC	TAGCCCTTCTTAGAGTGCC
*SgCPR1*	CGGCAGTCGGAAAGTTAAGA	GTCTGTGTACCGAAGAAGATTGA
*SgCPR2*	TGCGTTGTGGTCCTTGTT	GGCTCTGGCTCTTTGACTATC
*SgCYP102801*	GACGATGTCTACGAAGCGATAC	GTGGGTGACATTGAAGGAAGA
*SgEPH1*	CCGATGATACCGAAAGAGAAGG	GAACTTGCCGGCGAAATAATC
*SgEPH2*	GTGACCCACAAGCTCCATATT	CGGCGTAAACGTCGATATCTT
*SgEPH3*	CTTGGGATCGAGAAGGTGTTT	CACCAGCGCCTTGATTCTAT
*SgEPH4*	ACTATCGCGCTATTGACCTAAC	CAAATCGAGATCGCCGACTATAA
*SgUGT73-251-5*	TATGAGTCCGATGCTCCAATTC	GAGAGAAGAAGAGGGAGGAAGA
*SgUGT73-327-2*	AGAGAGTGAGAGAGAGCAAGAG	CAGCCACAGTGAGTGACAAA
*SgUGT73-348-2*	GAAGAAAGGACGAAGGGAAGAG	GCCGCAGTGGGTTAGAAA
*SgUGT73-251-6*	GAAAGTGAGGGAAGCCATAGAA	TATAGCTCTCTTCGCCGTCT
*SgUGT74-345-2*	CGACCATTCCATCGGCATATT	GCCTAGTGTTCTTCCCATCTTC
*SgUGT75-281-2*	GACTCGTCGGTCGTTTACATATC	ACGTGTGGCCACTGTTTAATA
*SgUGT85-269-1*	CCGATTGAAGTAGCGGAAGAA	CCTCAACGAGCTTCGGTATAAA
*SgUGT85-269-4*	CTCAATCCTCCGGTCTCATTC	GGGTCCAACGGTGTAGATTT
*SgUGT94-289-1*	CTGTTAACCTCGACGCCATTA	GCTGATCAGGAGAAGATGGAAG
*SgUGT94-289-2*	CCAAACACGGACCTACCTATT	GACCAACCGTCAGTGTAGTT
*SgUGT94-289-3*	CAGAGAAGATGTGCGGAAGAA	TCAGCGACCATCTCATCAAAC
*SgUBQ*	ATAAAAGACCCAGCACCACATTC	CCCTTGCCGACTACAACATCC
